# Supplementary material for: Anomalously bright single-molecule upconversion electroluminescence
Source: Nat Commun. 2024 Feb 23;15:1677. doi: 10.1038/s41467-024-45450-5 (PMC10891098; doi:10.1038/s41467-024-45450-5)
Supplement: Supplementary file 1 — Supplementary Information [file 41467_2024_45450_MOESM1_ESM.pdf]

Supplementary Information for

Anomalously Bright Single-Molecule Upconversion Electroluminescence

Yang Luo,[1,#] Fan-Fang Kong,[1,#] Xiao-Jun Tian,[1] Yun-Jie Yu,[1] Shi-Hao Jing,[1] Chao Zhang,[1] Gong Chen,[1,*] Yang Zhang,[1,2,3] Yao Zhang,[1,2,3] Xiao-Guang Li,[4] Zhen-Yu Zhang,[1,2,3] and Zhen-Chao Dong[1,2,3,*]

[1]*Hefei National Research Center for Physical Sciences at the Microscale and CAS Center for Excellence in Quantum Information and Quantum Physics, University of Science and Technology of China, Hefei, Anhui 230026, China*

[2]*School of Physics and Department of Chemical Physics, University of Science and Technology of China, Hefei, Anhui 230026, China*

[3]*Hefei National Laboratory, University of Science and Technology of China, Hefei 230088, China*

[4]*Institute for Advanced Study, Shenzhen University, Shenzhen, 518060, China*

[#]These authors contributed equally to this work.

*Corresponding authors. E-mail: gongchen@ustc.edu.cn, zcdong@ustc.edu.cn

Contents

Supplementary Note 1. Peak shift issue in the STML spectra of H_2Pc/3ML-NaCl/Au(111)...........2

Supplementary Note 2. Onset voltages of UCEL for H_2Pc/NaCl/Au(111)......................................3

Supplementary Note 3. More detailed discussions on the CI+CI UCEL mechanism.......................5

Supplementary Note 4. Energy requirements for the molecular transition from D_0^- to T_1...............8

Supplementary Note 5. Master equation theory for the single-molecule electroluminescence 10

Supplementary Note 6. Bias-dependent tip retraction and photon emission intensities on H_2Pc/3ML-NaCl/Au(111)...17

Supplementary Note 7. Simulated EL diagrams for some prototypical single-molecule systems..18

Supplementary Note 7.1 H_2Pc/3ML-NaCl/Au(111) with more detailed discussions 18

Supplementary Note 7.1.1 EL diagrams considering transient ground-state charged states 18

Supplementary Note 7.1.2 EL diagrams considering transient excited charged states ...20

Supplementary Note 7.1.3 Comparison with previous STML studies for the "same" H_2Pc/3ML-NaCl/Au(111) system 24

Supplementary Note 7.2 H_2Pc/3ML-NaCl/Ag(100)...25

Supplementary Note 8. Bias-dependent STML spectra for PtPc/NaCl/Au(111) 26

Supplementary Note 1. Peak shift issue in the STML spectra of H_2Pc/3ML-NaCl/Au(111)

The peak position in STML spectra is affected by several factors, including the Stark effect, photonic Lamb shift[1-3], and in the special case of H_2Pc/NaCl/Au(111), the splitting of the Q_x band for two differently orientated H_2Pc tautomers with respect to linear Moiré patterns resulting from the incommensurate NaCl and Au(111) lattices[4]. The Stark effect on the peak shift from 1.5 V to 1.7 V is believed to be negligible in the present system, as rationalized below. According to the paper by Imada *et al.*[2], the Stark effect was observed to cause a redshift of only ~1 meV when changing the voltage over a much wider range from –3 V to +1 V. In main-text Fig. 1b, there was a 2-meV blue-shift in the Q_x emission peak in the STML spectrum when the bias voltage was increased from 1.5 V to 1.7 V. This process is accompanied by an increase in the gap distance of ~0.15 nm to keep the tunneling current constant (see Supplementary Fig. 7(a)). Thus, the increases in the bias voltage and the gap distance have opposite effects on the electric field and are believed to largely cancel out with each other, leading to similar dc electric field strengths. Therefore, in the present system, the peak shift caused by the Stark effect is believed to be very minor, much smaller than 1 meV when the bias voltage is increased from 1.5 V to 1.7 V. Since such a small Stark shift has a negligible influence on the energy levels of molecular orbitals including HOMO and LUMO, the regulation of these molecular orbitals and related state energies by the electric field is not considered in our theoretical simulations.

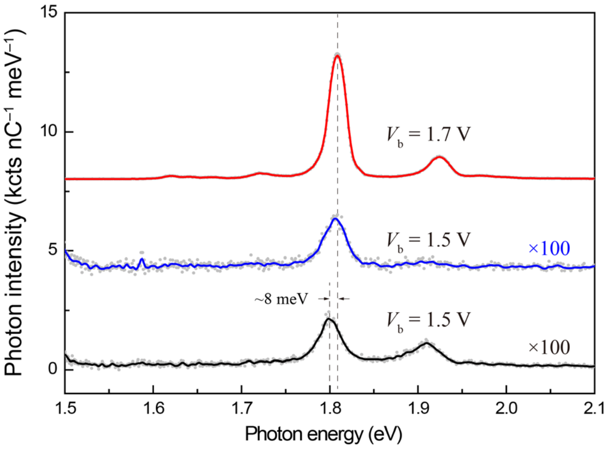

Supplementary Fig. 1: STML spectra acquired from H_2Pc/3ML-NaCl/Au(111) for three different experimental conditions. Black curve: 1.5 V, 30 pA, 300 s, blue curve: 1.5 V, 10 pA, 600 s, red curve: 1.7 V, 30 pA, 60 s. The black curve here might be obtained at a different position slightly off the molecular lobe. Spectral curves are offset for clarity.

Having excluded the possibility of the Stark effect being responsible for the 2-meV blue shift shown in main-text Fig. 1b, such a large blue shift is likely to originate from the photonics Lamb shift. Nevertheless, in addition to the typical dataset shown in main-text Fig. 1b, we also occasionally obtained STML spectra with abnormally large blue shifts (~8 meV) exemplified in the black curve of Supplementary Fig. 1 at 1.5 V. Such a large variation in the blue shift is unlikely to come from photonic Lamb shifts for a tip positioned at a given molecular position with similar changes in the tip height. In other words, the photonic Lamb shift may be responsible for the consistent blue shift of ~2 meV, while the anomalously large blue shift of ~8 meV is likely due to the splitting of the Q_x band for two differently orientated H_2Pc tautomers with respect to linear Moiré patterns resulting from the incommensurate NaCl and Au(111) lattices[4]. Such a phenomenon occurs when the tip is positioned at slightly different positions or tip positions are shifted during the STML measurements.

Supplementary Note 2. Onset voltages of UCEL for H_2Pc/NaCl/Au(111)

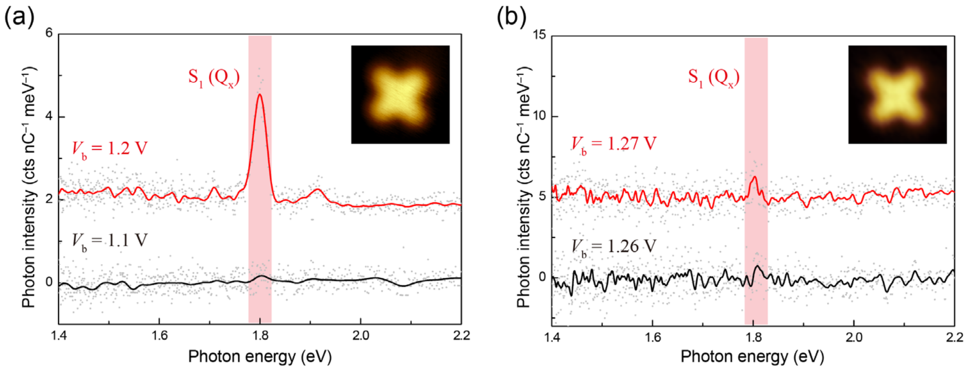

Supplementary Fig. 2: Onset voltages of UCEL for H_2Pc/NaCl/Au(111) with two different NaCl layer thicknesses. (a) STML spectra from a single H_2Pc molecule adsorbed on 2ML-NaCl/Au(111) at bias voltages (V_b) of 1.1 V and 1.2 V, respectively (50 pA, 600 s). (b) STML spectra from a single H_2Pc molecule adsorbed on 3ML-NaCl/Au(111) at bias voltages (V_b) of 1.26 V and 1.27 V, respectively (10 pA, 1200 s). Spectral curves are offset for clarity. The slightly increased onset voltage of UCEL for H_2Pc on 3ML-NaCl/Au(111) is possibly due to a slightly larger voltage drop in the thicker NaCl decoupling layer. The inset STM images in (a) and (b) (–0.7 V, 2 pA, 2.5 × 2.5 nm^2) show typical isolated molecular samples for the STML measurements in this work, illustrating the four-lobe molecular skeleton of H_2Pc. The STML spectra presented in the present work were acquired above the lobe.

Since the UCEL phenomenon can be observed at very small tunneling currents (typically less than 50 pA), the higher-order tunneling mechanism involving very large currents[5] in analogy to the

two-photon excitation process appears unlikely here. As a result, the involvement of an intermediate relay state in the nonlinear process becomes the most probable option. As shown in Supplementary Fig. 2, the UCEL onset voltage of a single H_2Pc molecule can be identified as ~1.2 eV on 2ML-NaCl/Au(111) and ~1.27 eV on 3ML-NaCl/Au(111), which agrees with the triplet state energy[6]. Similar phenomenon has been observed previously for UCEL from H_2Pc/NaCl/Ag(100) at negative bias. Therefore, the long-lifetime spin-triplet T_1 state is most likely to be the intermediate state for the excitation mechanism of anomalously bright single-molecule UCEL, similar to the previous UCEL mechanism for H_2Pc/NaCl/Ag(100) at negative bias[7].

We would like to note that characterizing key intermediate relay states, such as charged states and the T_1 state, as well as the alignment between molecular orbitals and the Fermi energy levels of the electrodes, is indeed a challenging endeavor. In cases where the bias drop in the NaCl layers can be considered negligible, the energy level alignment between the frontier molecular orbitals and the Fermi level of the substrate can be characterized through differential conductance measurements. For instance, the presence of two peaks in main-text Fig. 1c corresponds to the energy difference between the HOMO (LUMO) and the Fermi level of the substrate. When the HOMO (or LUMO) state becomes energetically accessible, the molecule can transiently become charged, and these peaks signify the existence of charged states. Additionally, the presence of charged states can also be detected through electroluminescence spectra, as demonstrated by the cationic emission in Supplementary Fig. 9 for H_2Pc/NaCl/Au(111) (as also reported by Rai *et al.*[8]).

In contrast, the characterization of the triplet relay state is much more challenging. In our model, its existence is mainly based on the agreement between the experimentally detected onset voltages of single-molecule UCEL and the energy of the T_1 triplet state. Nevertheless, phosphorescence from the triplet state was observed for the PtPc/NaCl/Ag(100) system by Grewal[9], but not in the PtPc/NaCl/Au(111) and H_2Pc/NaCl/Au(111) systems, probably due to either larger optical loss of the gold substrate or very weak spin-orbit coupling for H_2Pc. On the other hand, the characterization of the existence of the spin-triplet state experimentally via differential conductance is also highly challenging at the single-molecule level, probably due to very weak signals and resultant poor signal-to-noise ratio. According to a review article by Hipps and Mazur[10], the IES induced conductance increment in the *dI/dV* spectrum of the present experiment is estimated to be on the order of a few percent over the value before the IES channel is switched on. As shown in main-text

Figs. 1c and 4a, these values are too low to be resolved at the stable tunneling conditions adopted here, with the tunneling current on the order of picoamperes for the single molecules on the NaCl-covered Au(111). Consequently, we did try hard but failed to detect inelastic features in the *dI/dV* spectra, probably due to poor signal-to-noise ratios.

Supplementary Note 3. More detailed discussions on the CI+CI UCEL mechanism

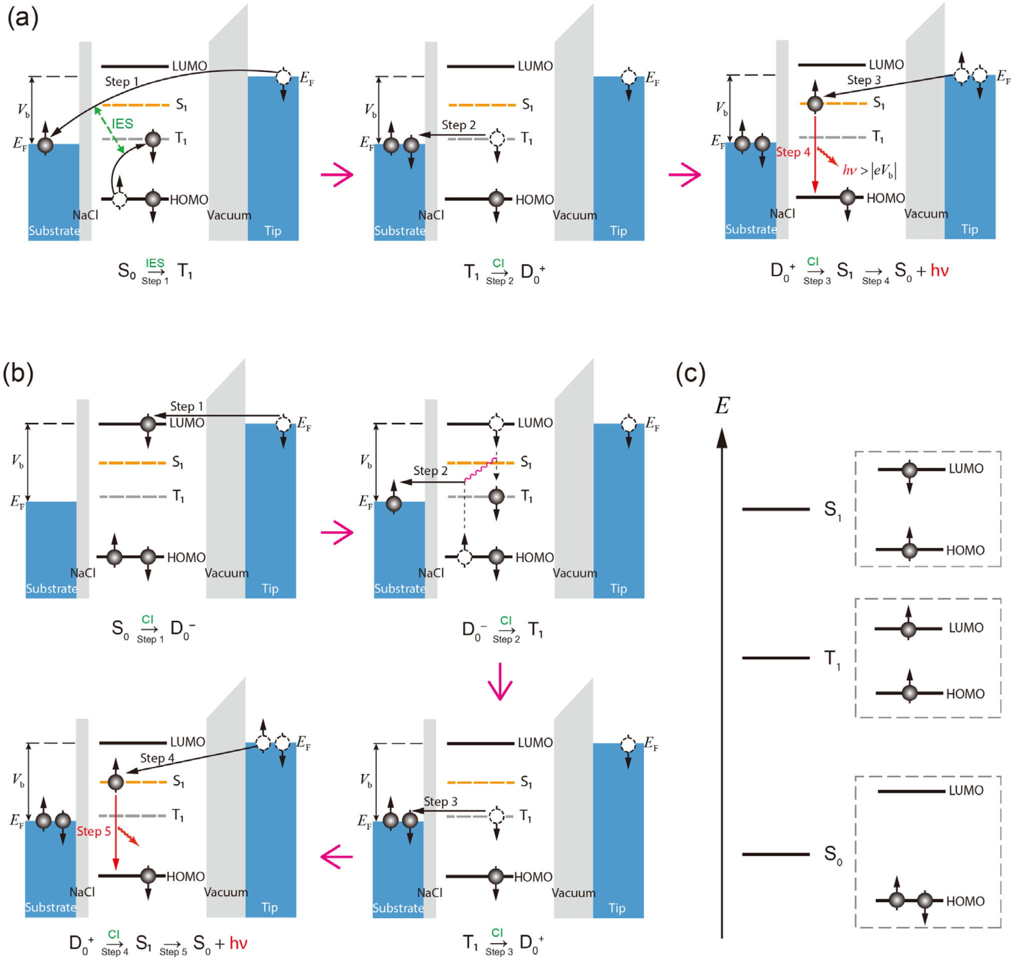

Supplementary Fig. 3: More details on the schematic diagrams of contrasting single-molecule UCEL mechanisms. (a) IES+CI UCEL mechanism. (b) CI+CI UCEL mechanism. (c) Electronic configurations for three neutral molecular states. Here the dotted circle with an arrow refers to the original position of the electron before a tunneling event or an electronic transition. We note that the schematic diagrams shown in (a,b) are plotted in a way that can better reflect how electrons tunnel in or out of a molecular orbital/state under an applied bias voltage. The electronic configurations for the three molecular states of a neutral molecule, i.e., S_0, T_1, and S_1, are actually those shown in (c). The energy levels shown in (a,b) refer to those of a neutral molecule.

In order to demonstrate the excitation processes of the IES+CI and CI+CI mechanisms more

clearly, we replot each step in main-text Fig. 2 separately (Supplementary Fig. 3). Additionally, further discussions are provided regarding the two distinct UCEL mechanisms. When $V_b < 1.6$ V (corresponding to the IES+CI regions in main-text Fig. 2b), the electron tunneling from the tip to the LUMO is energetically forbidden; therefore, the tunneling currents are dominated by the direct tunneling from the tip through the molecule-NaCl complex to the substrate. During the tunneling process, the molecule can be excited from the S_0 to T_1 via inelastic electron–molecule scattering, in which the tunneling electron has to exchange with one of the HOMO electron (which has an opposite spin, see Supplementary Fig. 5 and discussions there). Then the metastable electron in T_1 can first tunnel to the substrate, enabling a subsequent electron to directly inject from the tip into the S_1 state, which finally emits a photon. For the above IES+CI mechanism, the spin-triplet state T_1 plays an important role, since its long lifetime (~130 μs in dilute solution[6]) enables it to serve as an effective relay state to accumulate the energy of two successive tunneling electrons. A similar mechanism was also proposed to explain the UCEL from H_2Pc/NaCl/Ag(100), but at an opposite bias polarity.

Given its scattering nature (i.e., with a very short electron–molecule collision time), IES is believed to be an inefficient excitation mechanism and thus significantly limits the UCEL efficiency. Conceptually, the UCEL efficiency can be significantly improved if both the excitations from S_0 to T_1 and from T_1 to S_1 can be realized by sequential CI steps (rather than IES). As schematically illustrated in Supplementary Fig. 3(b), this mechanism, named CI+CI, is responsible for the highly efficient UCEL phenomenon observed for H_2Pc/NaCl/Au(111) (corresponding to the CI+CI regions in main-text Figs. 1c and 2b). Specifically, when the Fermi level of the tip lies above the LUMO of the molecule (i.e., when $V_b \geq 1.6$ V), the molecule can be excited from the ground state S_0 to the singlet excited state S_1 by four sequential CI steps, with the help of the long-lifetime spin-triplet state and two transient radical states. In the first CI step, an electron in the tip tunnels to the LUMO, generating a transient molecular anion D_0^-. In the presence of this LUMO electron, the electron energy in the original molecular "HOMO" can shift upwards above the Fermi level of the metal substrate owing to the mutual Coulombic interaction, thus allowing the "HOMO" electron to tunnel to the substrate. In other words, a hole in the substrate can inject into the up-shifted molecular "HOMO", leaving the molecule in the T_1 neutral intermediate state, but not in the S_1 state due to the energy conservation principle. The generation of T_1 requires $\phi_e \geq E_{T_1}$, where ϕ_e is the electron

injection barrier defined by the energy difference between the molecular LUMO and the Fermi level of the substrate.

Then the metastable electron in T_1, which lies above the Fermi level of the substrate (since $E_{T_1} > \phi_h$, where ϕ_h is the hole injection barrier defined by the energy difference between the molecular HOMO and the Fermi level of the substrate), is able to tunnel to the substrate, leaving behind a hole in the molecule. As a result, the original LUMO of the neutral molecule becomes the "LUMO" of a transient cation D_0^+, but its energy is significantly lowered due to the attractive Coulomb interaction[11,12]. Thus, another electron in the tip can then inject into this lowered "LUMO" and bring the molecule back to neutral but at the excited state S_1 without violating the energy conservation[7]. In other words, the second tunneling electron enables the upconversion from T_1 to S_1 via a transient cationic state through two sequential carrier injection steps.

We note that the CI+CI UCEL mechanism proposed in this work involving sequential carrier injection steps into a single molecule is quite different from the triplet–polaron-interaction-induced upconversion from triplet to singlet reported previously in OLEDs relying on intermolecular wave function overlaps within the framework of triplet–charge interactions (e.g., in Ref. 13 by Obolda *et al.*). In our model, the triplet–charge interaction is only one of the steps ($T_1 \rightarrow D_0^+$, step 3 in main-text Fig. 2b) for the upconversion process ($T_1 \rightarrow S_1$), whose realization requires an additional cation–charge interaction step ($D_0^+ \rightarrow S_1$, step 4 in main-text Fig. 2b); While in the paper by Obolda *et al.*, the upconversion from T_1 to S_1 is accomplished via one step of triplet–polaron interactions (e.g., $T_1 + D_0^\pm \rightarrow D_0^\pm + S_1$, or $T_1 + P^\pm \rightarrow P^\pm + S_1$). We would like to emphasize here, since the excitations from S_0 to T_1 and from T_1 to S_1 occur within a single molecule in our study (without intermolecular interactions), the observation of anomalously bright UCEL imposes critical requirements for the energy level alignment at the interfaces (see the red triangular area in main-text Fig. 3b) so that the amazing transitions between different charge states (neutral, cationic and anionic states) can occur under the condition of involving only the carrier injection steps. That is probably why such anomalously bright single-molecule UCEL has not been observed before and the underlying microscopic mechanism is so difficult to discover. In addition, due to the more complex interactions in ensemble molecular systems, it remains unclear where the energy of triplet–polaron interaction induced upconversion comes from: phonon or electric field effect, and how electric field provides

the driving force for the upconversion? In this regard, we would like to emphasize that single-molecule UCEL studies offer unique advantages: one can precisely control the local environment of individual molecules as well as the energy level alignment at the interfaces, thus clearly revealing the microscopic mechanisms of electroluminescence and UCEL at the single-molecule level that are difficult to unravel in ensemble studies.

Supplementary Note 4. Energy requirements for the molecular transition from D_0^- to T_1

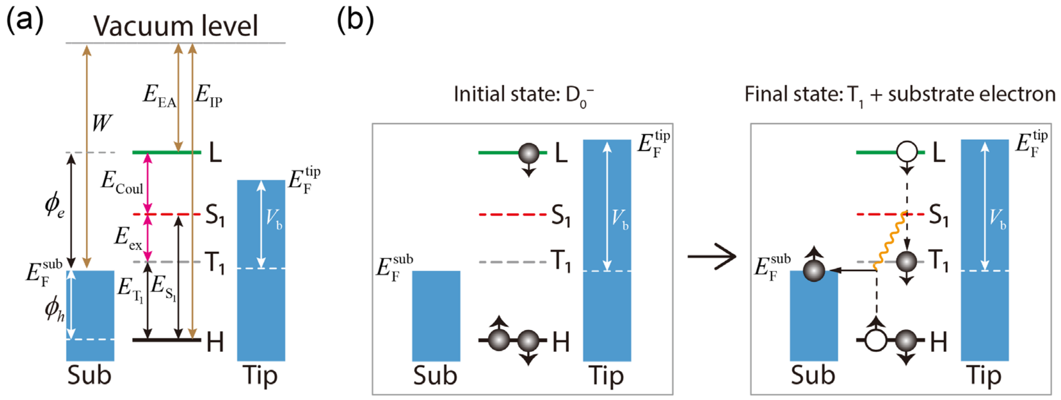

Supplementary Fig. 4: Schematic illustrating the energy requirements for the transition from D_0^- to T_1. (a) Energy level diagram of a biased single-molecule junction when molecular orbitals are outside the bias window. The vacuum level is taken as the energy reference. Here the molecular orbitals and energy states refer to a closed-shell neutral molecule without charging. E_{T_1} (E_{S_1}) is the optical gap of the lowest excited spin-triplet (spin-singlet) state, ϕ_e (ϕ_h) is the electron (hole) injection barrier defined as the energy difference between the molecular LUMO (HOMO) and the Fermi level of the substrate (E_F^{sub}), V_b is the bias voltage, E_{EA} is the molecular electron affinity, E_{IP} is the molecular ionization potential, E_{Coul} is the intramolecular Coulombic interaction energy, E_{ex} is the electron exchange energy, W is the work function of the substrate, and E_F^{tip} (E_F^{sub}) refers to the Fermi level of the tip (substrate). Here $E_F^{sub} = -W$ and $E_F^{tip} = -W + eV_b$. (b) Schematic showing the molecular transition from D_0^- to T_1 induced by CI at the positive bias.

Here we consider quantitatively the energy requirement for the second step of the CI+CI mechanism illustrated in main-text Fig. 2b, where the molecule is transformed from the anionic state D_0^- to the neutral spin-triplet state T_1. Supplementary Fig. 4 illustrates the energy level diagram of the single-molecule junction and the molecular transition $D_0^- \rightarrow T_1$ via the CI mechanism. We believe although it is a simplified model, it captures the main physics for the excitation process of single-molecule electroluminescence. According to the energy conservation law, for this CI induced transition to occur, the energy of the initial configuration (with the molecule in the D_0^- state, left

panel in Supplementary Fig. 4(b)) should be higher than the total energy of the final configuration (with the molecule in the T_1 state and one electron at the Fermi level of the substrate, right panel in Supplementary Fig. 4(b)), namely, $E_{D_0^-}^{total} \geq E_{T_1}^{total} + E_F^{sub}$. Here $E_{D_0^-}^{total}$ is the total energy of the D_0^- state, $E_{T_1}^{total}$ is the total energy of the T_1 state and E_F^{sub} is the Fermi level of the substrate. Since $E_{T_1}^{total} = E_{S_0}^{total} + E_{T_1}$ and $E_{S_0}^{total} - E_{D_0^-}^{total} = E_{EA}$, where $E_{S_0}^{total}$ is the total energy of the S_0 state and E_{T_1} is the excitation energy of T_1. The above inequality about the energy condition can be simplified to $\phi_e \geq E_{T_1}$, where $\phi_e = W - E_{EA}$ is the electron injection barrier (see Supplementary Fig. 4(a)). This criterion is satisfied for the H_2Pc/3ML-NaCl/Au(111) system since $\phi_e \approx 1.6$ eV is larger than the spin-triplet state excitation energy $E_{T_1} \approx$ 1.2 eV, as can be inferred from the *dI*/*dV* data shown in main-text Fig. 1c.

The transition $D_0^- \to T_1$ induced by CI at the positive bias can also be understood more intuitively using an energy shifting picture, as discussed in main-text Fig. 2b. In the presence of the LUMO electron, the electron energy in the original molecular "HOMO" can shift upwards above the Fermi level of the metal substrate owing to the mutual Coulombic interaction, thus allowing the "HOMO" electron to tunnel to the substrate (step 2). In other words, a hole in the substrate can inject into the up-shifted molecular "HOMO", leaving the molecule in the T_1 neutral intermediate state. The amount of the "HOMO" up-shifting ΔE_{HOMO} corresponding to $D_0^- \to T_1$ is determined by the intramolecular Coulomb interaction energy E_{Coul} and the electron exchange energy E_{ex}, as schematically illustrated in Supplementary Fig. 4(a). For H_2Pc/3ML-NaCl/Au(111) system, the HOMO-LUMO gap is estimated to be 2.7 eV (considering the onset voltage of the HOMO and LUMO peaks in the *dI*/*dV* spectrum shown in main-text Fig. 1c), the singlet and triplet excitation energies are ~1.8 eV and ~1.2 eV, respectively. Thus we can estimate the intramolecular Coulombic interaction energy to be E_{Coul} ~0.9 eV and the electron exchange energy to be E_{ex} ~0.6 eV. Therefore, the HOMO is up-shifted by $\Delta E_{HOMO} \approx E_{Coul} + E_{ex} \approx 1.5$ eV, a quantity large enough to shift the new "HOMO" above the Fermi level of the substrate. Note that the molecular energy levels can undergo significant modifications when the molecule is located within a metal nanogap (see, e.g., Refs. 14,15). The charging energy or Coulomb repulsion energy of an isolated molecule in the gas phase typically amounts to a few electronvolts. However, within a metal nanogap, this value can

be reduced to below 1 eV due to several effects, including the influence of the image potential generated by the substrate and the hybridization with the metal electrodes.

Using similar considerations on the energy-level alignment, one can also justify the occurrence of the upconversion process from T_1 to S_1 via a transient cationic state through two sequential carrier injection steps for the H_2Pc/3ML-NaCl/Au(111) system. Since $E_{T_1} > \phi_h$ ($\phi_h \approx 1.1$ eV, main-text Fig. 1c), the metastable electron in T_1 can tunnel to the substrate, leaving behind a transient cation. Due to the attractive Coulomb interaction, the "LUMO" of the cation can be significantly lowered by $\Delta E_{\mathrm{LUMO}} \approx E_{\mathrm{Coul}} \approx 0.9$ eV. Thus, another electron in the tip can easily inject into this "LUMO" for a bias above 1.6 V and bring the molecule back to neutral but at the excited state S_1 without violating the energy conservation.

Supplementary Note 5. Master equation theory for the single-molecule electroluminescence

For an electronically decoupled molecule, the single-molecule junction can be considered as a double-barrier tunnel junction. For single molecules deposited on ultrathin NaCl films, it was found that the voltage drops nearly exclusively in the vacuum gap between the tip and the molecule[16]. Therefore, in our model we shall assume that the molecular levels are pinned to the substrate. At low bias, when molecular orbitals lie outside the energy window spanned by the Fermi levels of the tip and the substrate, no electrons or holes can be injected into the neutral molecule. When the bias voltage is increased in the positive direction, the Fermi level of the tip gradually surpasses the unoccupied molecular orbitals and electron injections into the neutral molecule are accessible. Conversely, at sufficiently large negative voltages, hole injections into (or electron extraction from) the occupied molecular orbitals can become possible. In the electroluminescence process, the molecule is often excited to the neutral excited states via the intermediate cationic or anionic states. To model single-molecule electroluminescence, one should properly consider these carrier injection (CI) processes as well as several other excitation and relaxation processes, as detailed below. In this work, we focus on the molecular excitation process and the central physical quantity to calculate is the excitation efficiency η_{ex} to convert the energy of tunneling electrons to molecular excitons, as detailed below.

The excitation and decay kinetics of a single molecule in a biased tunneling junction can be

described by the master equation[7,17,18]

$$\frac{d}{dt}\rho_{ab} = -i\left[H_{\text{mol}}, \rho\right]_{ab} - \delta_{ab}\sum_{c}\left[\gamma_{a\to c}\rho_{aa} - \gamma_{c\to a}\rho_{cc}\right] - (1-\delta_{ab})\gamma_{ab}^{\text{ph}}\rho_{ab}, \tag{1}$$

where ρ is the molecular density operator, δ_{ab} is a Kronecker delta symbol, H_{mol} is the free Hamiltonian of the single molecule, *a*, *b* and c represent the molecular quantum states. The transition rate from *a* to *c* includes four terms:

$$\gamma_{a\to c} = \gamma_{a\to c}^{\text{CI}} + \gamma_{a\to c}^{\text{IES}} + \gamma_{a\to c}^{\text{rad}} + \gamma_{a\to c}^{\text{non-rad}},$$

which corresponds to the CI induced transition, the IES induced transition, the radiative decay, and non-radiative decay, respectively. In this paper, five molecular states are considered, including the neutral ground state S_0, the lowest excited spin-triplet T_1 and spin-singlet S_1 states, as well as the ground cationic D_0^+ and anionic D_0^- states. The electroluminescence from charged molecular states can also be included in the master equation straightforwardly when these excited charged states are considered in the master equation, as exemplified later in Supplementary Note 7.1.2 for the cationic emission.

Two basic excitation mechanisms are taken into account in our model, namely, the sequential charge carrier injection (CI) from the metal electrodes to the molecule and the inelastic electron–molecule scattering (IES)[7,10,19,20]. Each CI step changes the net charge of the molecule by ±*e* and requires a proper energy level alignment to occur. For example, the excitation $S_0 \to S_1$ can be induced by two sequential CI steps via an intermediate transient charged state ($S_0 \to D_0^{\pm} \to S_1$) (see the right panel of main-text Fig. 3c and Ref. 7). In the CI regime, the tunneling induced molecular transition rates are

$$\gamma_{D_0^+\to a}^{\text{CI}} = M_a \times \left\{\Gamma_{\text{tip}} \times n_{\text{F}}^{\text{tip}}(-\phi_{\text{h}} + \mathcal{E}_a) + \Gamma_{\text{sub}} \times n_{\text{F}}^{\text{sub}}(-\phi_{\text{h}} + \mathcal{E}_a)\right\},$$
$$\gamma_{a\to D_0^+}^{\text{CI}} = N_a \times \left\{\Gamma_{\text{tip}} \times [1 - n_{\text{F}}^{\text{tip}}(-\phi_{\text{h}} + \mathcal{E}_a)] + \Gamma_{\text{sub}} \times [1 - n_{\text{F}}^{\text{sub}}(-\phi_{\text{h}} + \mathcal{E}_a)]\right\},$$
$$\gamma_{D_0^-\to a}^{\text{CI}} = M_a \times \left\{\Gamma_{\text{tip}} \times [1 - n_{\text{F}}^{\text{tip}}(\phi_{\text{e}} - \mathcal{E}_a)] + \Gamma_{\text{sub}} \times [1 - n_{\text{F}}^{\text{sub}}(\phi_{\text{e}} - \mathcal{E}_a)]\right\},$$
$$\gamma_{a\to D_0^-}^{\text{CI}} = N_a \times \left\{\Gamma_{\text{tip}} \times n_{\text{F}}^{\text{tip}}(\phi_{\text{e}} - \mathcal{E}_a) + \Gamma_{\text{sub}} \times n_{\text{F}}^{\text{sub}}(\phi_{\text{e}} - \mathcal{E}_a)\right\},$$

where a represents one of the three neutral molecular states (i.e., $a = S_0, T_1$ or S_1), $\mathcal{E}_a = E_a^{\text{total}} - E_{S_0}^{\text{total}}$, referring to the excitation energy corresponding to the transition $S_0 \to a$, ϕ_{e} (ϕ_{h}) is the electron (hole) injection barrier, $n_{\text{F}}^{\text{tip}}(n_{\text{F}}^{\text{sub}})$ is the Fermi distribution function of the tip (substrate), and Γ_{tip} (Γ_{sub}) represents the bare tunneling rate between the molecule and the tip (substrate). In the above expressions of the CI induced transition rate, the spin multiplicities of the

neutral and charged states have been taken into account via the parameters M_a and N_a; Specifically $M_{\mathrm{S_0}}=1$, $M_{\mathrm{T_1}}=3/2$, $M_{\mathrm{S_1}}=1/2$, $N_{\mathrm{S_0}}=2$, $N_{\mathrm{T_1}}=1$, and $N_{\mathrm{S_1}}=1$[21]. To calculate the electric current (see below), we also define the electron tunneling rates from the tip to the molecule

$$\begin{aligned}
\gamma^{\mathrm{CI,tip}}_{\mathrm{D_0^+}\to a} &= M_a \times \Gamma_{\mathrm{tip}} \times n_{\mathrm{F}}^{\mathrm{tip}}(-\phi_{\mathrm{h}} + \mathcal{E}_a), \\
\gamma^{\mathrm{CI,tip}}_{a\to\mathrm{D_0^+}} &= N_a \times \Gamma_{\mathrm{tip}} \times [1 - n_{\mathrm{F}}^{\mathrm{tip}}(-\phi_{\mathrm{h}} + \mathcal{E}_a)], \\
\gamma^{\mathrm{CI,tip}}_{\mathrm{D_0^-}\to a} &= M_a \times \Gamma_{\mathrm{tip}} \times [1 - n_{\mathrm{F}}^{\mathrm{tip}}(\phi_{\mathrm{e}} - \mathcal{E}_a)], \\
\gamma^{\mathrm{CI,tip}}_{a\to\mathrm{D_0^-}} &= N_a \times \Gamma_{\mathrm{tip}} \times n_{\mathrm{F}}^{\mathrm{tip}}(\phi_{\mathrm{e}} - \mathcal{E}_a).
\end{aligned}$$

Compared with CI mechanism, the IES mechanism does not change the net charge of the molecule and is an inefficient excitation mechanism given the very short electron–molecule collision time[22]. It involves complex many-body interactions between the tunneling electrons and the molecule and an accurate evaluation can be quite demanding. In this work, the IES induced molecular excitation rates are estimated based on the tunneling current directly between the tip and the substrate as well as the IES efficiencies. More specifically,

$$\gamma^{\mathrm{IES}}_{S_0\to T_1} = \eta^{\mathrm{exch}}_{\mathrm{IES}} \times \gamma^{\mathrm{IES}}_{\mathrm{tip}\leftrightarrow\mathrm{sub}},\ \gamma^{\mathrm{IES}}_{S_0\to S_1} = \eta^{\mathrm{dir}}_{\mathrm{IES}} \times \gamma^{\mathrm{IES}}_{\mathrm{tip}\leftrightarrow\mathrm{sub}},\ \gamma^{\mathrm{IES}}_{T_1\to S_0} = \eta^{\mathrm{exch}}_{\mathrm{IES}} \times \gamma^{\mathrm{IES}}_{\mathrm{tip}\leftrightarrow\mathrm{sub}},\ \gamma^{\mathrm{IES}}_{S_1\to S_0} = \eta^{\mathrm{dir}}_{\mathrm{IES}} \times \gamma^{\mathrm{IES}}_{\mathrm{tip}\leftrightarrow\mathrm{sub}}.$$

Here $\eta^{\mathrm{dir}}_{\mathrm{IES}}$ ($\eta^{\mathrm{exch}}_{\mathrm{IES}}$) is the portion of tunneling electrons that excite the molecule via the non-spin-flip (spin-flip) process and $\gamma^{\mathrm{IES}}_{\mathrm{tip}\leftrightarrow\mathrm{sub}}$ is the tunneling rate between the tip and substrate in the IES regime. Note the tunneling induced molecular deexcitation processes are also considered. For simplicity, the IES efficiencies are treated as constants. It should be noted that the IES mechanism mainly originates from the collision between the molecule and the electrons that directly tunnels between the two electrodes. Thus, the IES mechanism becomes important when carrier injections through molecular orbitals are energetically prohibited. This requires both the HOMO and LUMO are outside the bias window (molecular orbitals other than the HOMO and LUMO are neglected for simplicity). In this situation we set $\gamma^{\mathrm{IES}}_{\mathrm{tip}\leftrightarrow\mathrm{sub}} = I_e/e$ where I_{e} is the electric current and e is the electron charge.

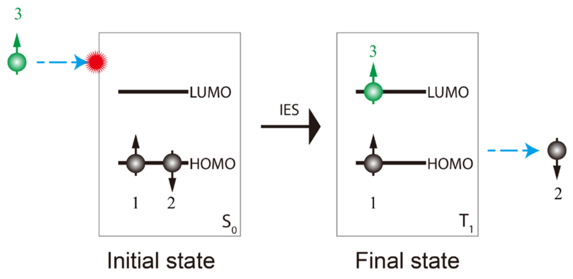

Supplementary Fig. 5: Schematic illustrating the spin-exchange inelastic scattering between an incident electron and the molecule. The molecule is excited from the singlet ground state (S_0) to the triplet state (T_1) after the scattering process. Different colors and labels are used for the incident and scattered electrons to show that the incident electron exchanges with the electron in the molecule.

In the following, we explain qualitatively why the direct excitation from S_0 to T_1 via the spin-exchange IES mechanism is not spin-forbidden. Since the IES induced transition rates involve complex many-body interactions and their accurate evaluation can be quite demanding, here we only try to rationalize its occurrence using simple arguments. Supplementary Fig. 5 schematically illustrates the spin-exchange inelastic scattering process between an incident electron and a single molecule where the molecule is excited from the singlet ground state to the spin-triplet state. For simplicity, only the two electrons in the HOMO are explicitly considered. The wavefunction for the initial state, consisting of the incident free electron and the ground-state molecule, can be written as

$$\begin{aligned}\Psi_i(r_1,r_2,r_3) &= \phi_{k_i}(r_3)\chi_\uparrow(3)\times\psi_{S_0}(r_1,r_2)\\ &= \phi_{k_i}(r_3)\chi_\uparrow(3)\times\varphi_H(r_1)\varphi_H(r_2)\frac{1}{\sqrt{2}}\left[\chi_\uparrow(1)\chi_\downarrow(2)-\chi_\downarrow(1)\chi_\uparrow(2)\right]\end{aligned}.$$

Here $\varphi_H(r_1)\varphi_H(r_2)$ and $\phi_{k_i}(r_3)$ are the spatial part of the wavefunctions for the S_0-state molecule and the incident electron (with k_i denoting the momentum), respectively, $\chi_{\uparrow/\downarrow}$ is the wavefunction for spin. The final state consists of the scattered free electron and the molecule in the triplet state, with its wavefunction written as

$$\begin{aligned}\Psi_f(r_1,r_2,r_3) &= \phi_{k_f}(r_2)\chi_\downarrow(2)\times\psi_{T_1}(r_1,r_3)\\ &= \phi_{k_f}(r_2)\chi_\downarrow(2)\times\frac{1}{\sqrt{2}}\left[\varphi_H(r_1)\varphi_L(r_3)-\varphi_L(r_1)\varphi_H(r_3)\right]\chi_\uparrow(1)\chi_\uparrow(3)\end{aligned}.$$

Here $\frac{1}{\sqrt{2}}\left[\varphi_H(r_1)\varphi_L(r_3)-\varphi_L(r_1)\varphi_H(r_3)\right]$ and $\phi_{k_f}(r_2)$ are the spatial part of the wavefunctions for the T_1-state molecule and the scattered electron (with k_f denoting the momentum), respectively. Note that to simplify our discussion, here we only consider one of the three possible configurations for the final triplet state. The Coulomb interaction between the incident electron and the two

electrons in the molecule is $V_{\text{e-e}} = \frac{e^2}{4\pi\epsilon_0}\left(\frac{1}{|r_1 - r_3|} + \frac{1}{|r_2 - r_3|}\right)$. With these notations defined, the transition amplitude for this spin-exchange inelastic scattering can be formally written as

$$\begin{aligned} T_{i\to f} &= \langle \Psi_f | V_{\text{e-e}} | \Psi_i \rangle \sim \int \varphi_{\text{H}}^*(r_1)\varphi_{\text{H}}(r_1)dr_1 \times \frac{e^2}{8\pi\epsilon_0}\int \phi_{k_f}^*(r_2)\varphi_{\text{L}}^*(r_3)\frac{1}{|r_2 - r_3|}\varphi_{\text{H}}(r_2)\phi_{k_i}(r_3)dr_2dr_3 \\ &= \frac{e^2}{4\pi\epsilon_0}\int \phi_{k_f}^*(r_2)\varphi_{\text{L}}^*(r_3)\frac{1}{|r_2 - r_3|}\varphi_{\text{H}}(r_2)\phi_{k_i}(r_3)dr_2dr_3 \end{aligned}.$$

We can see that the IES-induced excitation from S_0 to T_1 indeed requires a substantial overlap between the wavefunctions of the incident electron and the molecule, which ensures that the above integral is nonzero. Thus, this form of "exchange" interaction becomes effective only when the electron is very close to the electron cloud of the target molecule so that they can "exchange". Typically, this condition is met when the tip is positioned directly above the molecule, enabling tunneling electrons from the tip to traverse through the molecule. In fact, such a spin-exchange process via IES imposes no requirement on the strength of intramolecular spin-orbit coupling. As demonstrated previously for molecules lacking significant spin-orbit coupling such as H_2Pc and pentacene, their triplet transitions have been experimentally observed in inelastic electron tunneling spectroscopies within metal–insulator–metal tunnel junctions[6,19].

When through-orbital tunneling channels are accessible (i.e., when molecular orbitals enter the bias window), the IES-induced molecular excitation are still possible in principle, but strongly suppressed. This is because in this situation the tunneling currents are dominated by the tunneling through molecular orbitals (mainly limited by the tunneling rate between the molecule and the tip) and the percentage of tunneling currents directly from the tip to the substrate (or vice versa) is usually very low (the field emission at large voltages is not considered here). Such an excitation mechanism is termed weak-IES (or *w*-IES). In the *w*-IES regime, the molecular transition rates induced by the IES mechanism can be expressed as follows for excitation and decay processes:

$$\gamma_{\text{S}_0\to\text{T}_1}^{w\text{-IES}} = \eta_{\text{IES}}^{\text{exch}} \times \gamma_{\text{tip}\leftrightarrow\text{sub}}^{w\text{-IES}},\ \gamma_{\text{S}_0\to\text{S}_1}^{w\text{-IES}} = \eta_{\text{IES}}^{\text{dir}} \times \gamma_{\text{tip}\leftrightarrow\text{sub}}^{w\text{-IES}},\ \gamma_{\text{T}_1\to\text{S}_0}^{w\text{-IES}} = \eta_{\text{IES}}^{\text{exch}} \times \gamma_{\text{tip}\leftrightarrow\text{sub}}^{w\text{-IES}},\ \gamma_{\text{S}_1\to\text{S}_0}^{w\text{-IES}} = \eta_{\text{IES}}^{\text{dir}} \times \gamma_{\text{tip}\leftrightarrow\text{sub}}^{w\text{-IES}}.$$

We assume that once through-orbital tunneling are enabled, only 1% of the currents are produced by the direct tunneling between the tip and the substrate. Specifically, we set $\gamma_{\text{tip}\leftrightarrow\text{sub}}^{w\text{-IES}} = 0.01 \times I_e/e$.

By considering all the above transition rates, we can obtain the steady-state population of the singlet state $\rho_{\text{S}_1\text{S}_1}$ from the master equation Eq. (1), and then the photon emission intensity can be

evaluated via $I_{\text{ph}} = \rho_{\text{S}_1\text{S}_1} \times \gamma_{\text{S}_1\to\text{S}_0} \times \eta^{\text{rad}}$, where η^{rad} is the quantum efficiency for molecular emission in the STM cavity. For electroluminescence processes without laser irradiation, the off-diagonal elements of the density matrix are unimportant and thus the master equation can be reduced to the rate equation where only the diagonal elements of the density matrix are considered[21]. To quantify the electroluminescence properties, we can define the internal electron–photon conversion efficiency via $\eta_{\text{e-ph}} = I_{\text{ph}} / I_{\text{e}} = \rho_{\text{S}_1\text{S}_1} \times \gamma_{\text{S}_1\to\text{S}_0} \times \eta^{\text{rad}} / I_{\text{e}}$. When through-orbital tunneling is enabled, the total tunneling current can be expressed as $I_{\text{e}} = I_{\text{e}}^{\text{CI}} + I_{\text{e}}^{w\text{-IES}}$, where the predominant through-orbital tunneling current is given by

$$I_{\text{e}}^{\text{CI}} = \left(\rho_{\text{D}_0^+\text{D}_0^+} \sum_{a=S_0,T_1,S_1} \gamma_{\text{D}_0^+\to a}^{\text{CI,tip}} + \sum_{a=S_0,T_1,S_1} \rho_{aa} \gamma_{a\to\text{D}_0^-}^{\text{CI,tip}} \right) - \left(\sum_{a=S_0,T_1,S_1} \rho_{aa} \gamma_{a\to\text{D}_0^+}^{\text{CI,tip}} + \rho_{\text{D}_0^-\text{D}_0^-} \sum_{a=S_0,T_1,S_1} \gamma_{\text{D}_0^-\to a}^{\text{CI,tip}} \right).$$

We note that the radiation quantum efficiency η^{rad} depends on the plasmon–exciton coupling strength and the plasmonic response of the STM junction, and thus can vary greatly in different experiments. Nevertheless, for the same tip status, this quantity can be assumed to be constant. For simplicity, we neglect the quantum efficiency in the exciton radiation process and define the electron–exciton excitation efficiency as follows:

$$\eta_{\text{ex}} = \rho_{\text{S}_1\text{S}_1} \times \gamma_{\text{S}_1\to\text{S}_0} / I_{\text{e}},$$

which is determined by various current-induced molecular excitation processes. This is the central physical quantity in our model since it can reflect the bias-dependent electroluminescence behaviors under different energy-level alignments. The numerically evaluated electroluminescence (EL) diagrams for the molecular excitation map are two dimensional plots of η_{ex} as a function of the bias voltage V_{b} and the electron (or hole) injection barrier ϕ_{e} (or ϕ_{h}). Note that since the Stark shift is very small in the present system (see Supplementary Note 1) and has a negligible influence on the energy levels of molecular orbitals including HOMO and LUMO, the regulation of these molecular orbitals and related state energies by the electric field is not considered in our theoretical simulations.

The following parameters were used in the numerical simulations. We set the excitation energy of the triplet and singlet as $E_{\text{T}_1} = 1.2$ eV and $E_{\text{S}_1} = 1.8$ eV, and the decay rate of the triplet and singlet in the plasmonic nanocavity as $\gamma_{\text{T}_1\to\text{S}_0} = 10^7$ s^{-1} and $\gamma_{\text{S}_1\to\text{S}_0} = 10^{12}$ s^{-1}. The bare tunneling rate between the molecule and the substrate is set to be $\Gamma_{\text{sub}} = 10^9$ s^{-1}. In the IES regime, we set the bare tunneling rate from the molecule to the tip as $\Gamma_{\text{tip}} = 10^{10}$ s^{-1}, while in the CI regime, Γ_{tip} is set adaptively to give the total tunneling current. In both the IES and CI regimes, we set the tunneling

current as I_e = 30 pA, corresponding to typical experimental conditions. For the IES-induced excitation efficiencies, we set $\eta_{\mathrm{IES}}^{\mathrm{dir}} = 10^{-2}$ for the non-spin-flip excitation and $\eta_{\mathrm{IES}}^{\mathrm{exch}} = 10^{-3}$ for the spin-flip excitation.

The simulated EL diagram for the emission from the S_1 state at different bias polarities are shown in Supplementary Fig. 6 as a function of both bias and (electron or hole) carrier injection barriers, with two types of barrier heights relative to the triplet state energy. More detailed discussions about the excitation mechanisms in each region can be found in Supplementary Note 7. We note that the theoretical model proposed in this work only summarizes the most fundamental single-molecule electroluminescence behaviors, especially the spin-triplet mediated UCEL. In real experiments, other issues may also play a significant role in the single-molecule electroluminescence behaviors, for instance, carrier injection through higher-lying or lower-lying molecular orbitals[23], excited charged states[8,23,24], the voltage drops in the insulating layer[25], the finite linewidth of molecular orbitals, the energy-dependence of electronic density of states of the metal tip and substrate, the influence of the bias voltage on the IES excitation efficiency and the through-orbital CI rates, the influence of the dc Stark effect on the energies of molecular orbitals, etc. Since we have ignored all these issues, the numerically evaluated EL diagrams are simplified with respect to real experiments. For instance, although the simulated EL diagrams show distinctly different regions that clearly distinguish different excitation mechanisms for a given energy-level alignment, each region (corresponding to a particular dominant mechanism) is approximated by a constant excitation efficiency, as exemplified in Supplementary Fig. 6. This is because the influence of V_b, ϕ_e and ϕ_h on the excitation efficiency is not taken into account, while in real experiments the excitation efficiency inside each region could vary when these quantities are changed. In principle, all these factors can be included in a more generalized theoretical model, which will produce much richer EL diagram. As an example, discussions considering the transient excited cationic state and resultant cationic emission is presented in Supplementary Note 7.1.2.

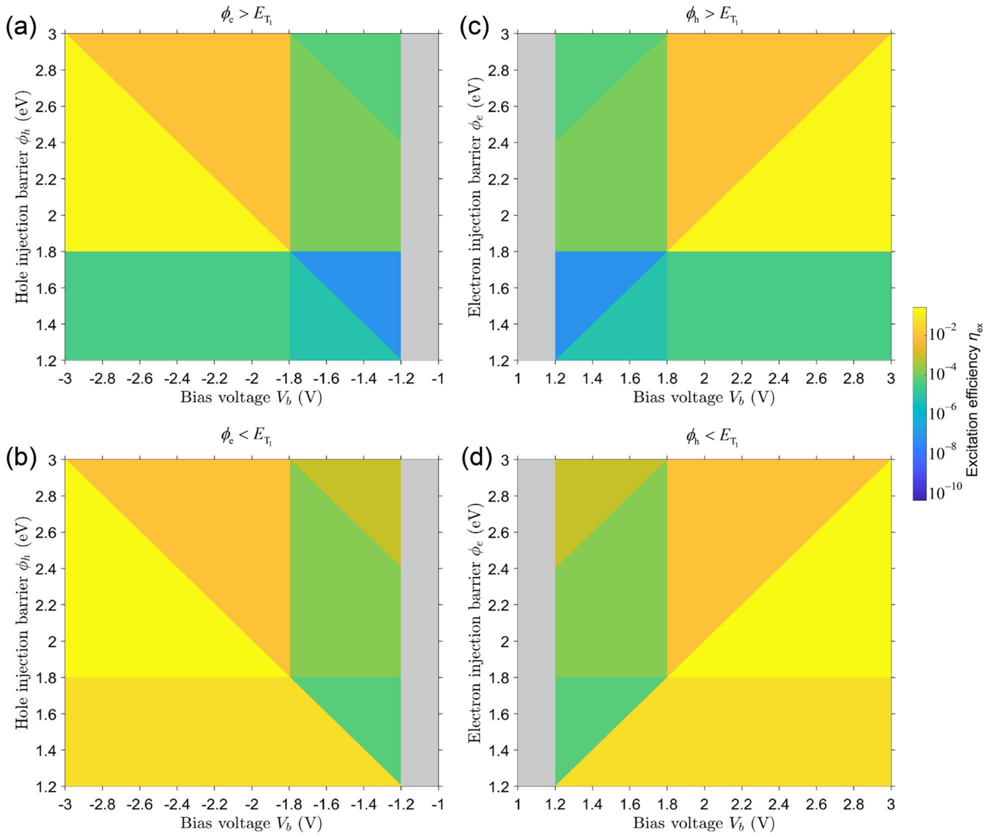

Supplementary Fig. 6: Simulated EL diagrams from the neutral excited state S_1 as a function of both bias voltages and carrier injection barriers for two types of barrier heights relative to the triplet state energy. (a, b) Excitation EL diagrams at negative bias for two different electron injection barriers: $\phi_e = 1.6$ eV (a, $\phi_e > E_{T_1}$) and $\phi_e = 1.0$ eV (b, $\phi_e < E_{T_1}$). (c, d) Excitation EL diagrams at positive bias for two different hole injection barriers: $\phi_h = 1.6$ eV (c, $\phi_h > E_{T_1}$) and $\phi_h = 1.0$ eV (d, $\phi_h < E_{T_1}$).

Supplementary Note 6. Bias-dependent tip retraction and photon emission intensities on H_2Pc/3ML-NaCl/Au(111)

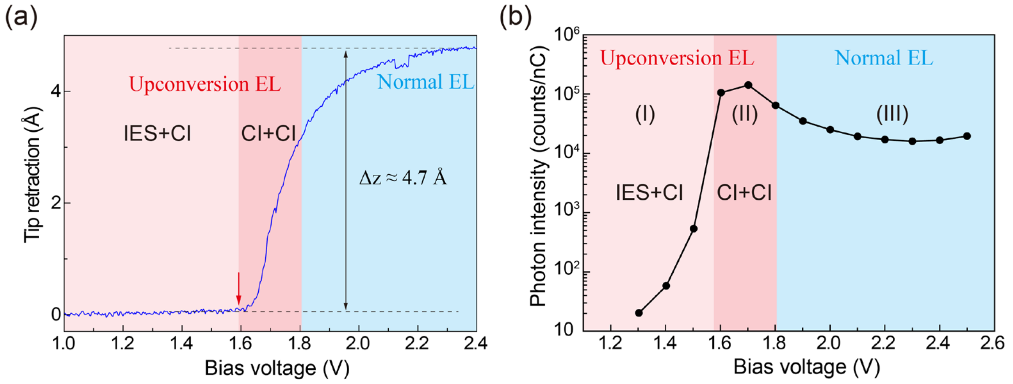

Supplementary Fig. 7: Bias-dependent tip retraction and electroluminescence intensities. (a) Vertical tip

retraction (Δz) as a function of the bias voltage (V_b = 1.0–2.4 V) measured at a setpoint current of 2 pA above a single H_2Pc molecule on the 3ML-NaCl/Au(111) substrate. (b) Bias-dependent intensity integrated over the Q_x peak for a single H_2Pc molecule on the 3ML-NaCl/Au(111) substrate. It can be seen that as the molecular LUMO enters the bias window above $V_b \approx 1.6$ V, the tip–molecule distance increases to ensure a constant current for the feedback loop. (The observed tip retraction of ~470 pm in Supplementary Fig. 7(a) is quite large, which reflects a big change in the electron tunneling probability up to several orders of magnitude when molecular LUMO states are available.) The increased tip–molecule distance can decrease the quantum efficiency of molecular emission in plasmonic nanocavities[1,26] and reduce the electroluminescence intensity, as illustrated in main-text Fig. 1c when $V_b \geq 1.8$ V. Note that the appearance of the small pit at about 2.15 V is likely to correlate with the hydrogen tautomerization event within a single H_2Pc molecule. We would like to note that although the photon intensity in STM induced molecular emission can be affected by the tip–molecule distance, the CI+CI UCEL mechanism we propose here is essentially correct and reliable. As shown in (a), the tip–molecule distance is almost constant below ~1.6 V. However, the UCEL intensity jumps by over two orders of magnitude as the excitation mechanism changes from IES+CI to CI+CI at ~1.6 V (main-text Fig. 1c). This very huge increase in UCEL intensities cannot be explained by the difference in the local electromagnetic enhancement effect associated with the tip–molecule distances.

Supplementary Note 7. Simulated EL diagrams for some prototypical single-molecule systems

Supplementary Note 7.1 H_2Pc/3ML-NaCl/Au(111) with more detailed discussions

Supplementary Note 7.1.1 EL diagrams considering transient ground-state charged states

Supplementary Fig. 8 schematically illustrates all the excitation processes associated with the EL diagram shown in main-text Fig. 3b, with the dominant excitation mechanism at each region schematically shown around the EL diagram. For instance, in the regions labeled as "IES+CI", although both the IES+CI and the IES+IES mechanisms coexist, the latter is not shown due to its much weaker efficiency[7]. The black horizontal dashed line in Supplementary Fig. 8 corresponds to the special case of the H_2Pc/3ML-NaCl/Au(111) system in the present work. In the simulated EL diagram, the horizontal and vertical boundaries dividing different "phases" (or different excitation mechanisms) correspond to either the triplet or singlet excitation energies. Besides, there are two boundary lines that are $45°$ with respect to the axes: $\phi_e = eV_b$ and $\phi_e = eV_b + E_{T_1}$. The boundary line $\phi_e = eV_b$ determines whether the through-orbital tunneling is allowed. That is, the electron injection from the tip to S_0 (i.e., $S_0 \to D_0^-$) is possible only below this line. The other boundary line $\phi_e = eV_b + E_{T_1}$ is correlated with the transition from T_1 to D_0^-. That is, the electron injection from

the tip to T₁ (i.e., $T_1 \rightarrow D_0^-$) is possible only below this line. We also note that depending on the value of the electron injection barrier ϕ_e, the IES+CI mechanism in the plot splits into three regions with different molecular excitation efficiencies via either a transient cationic state or a transient anionic state or both.

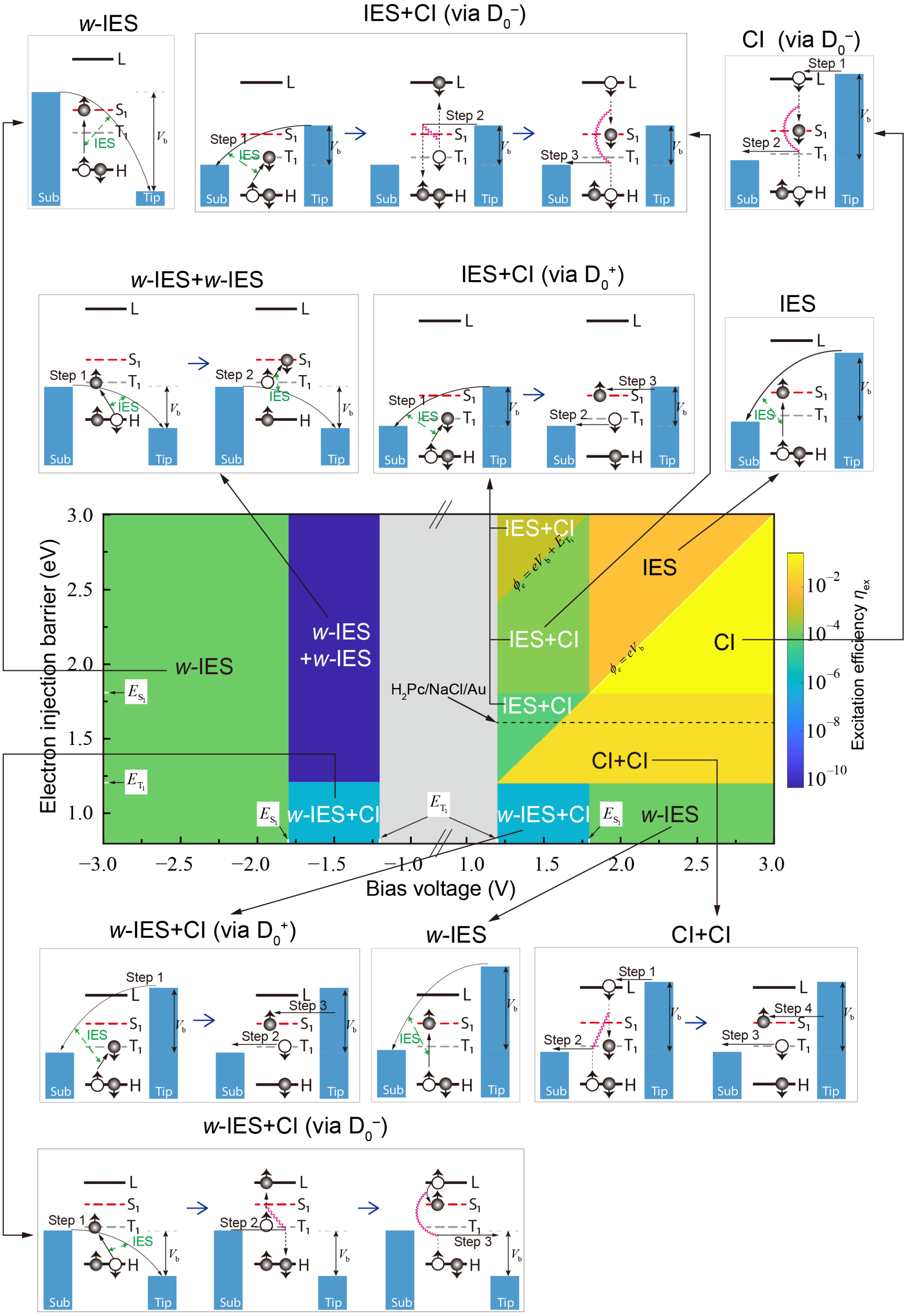

Supplementary Fig. 8: Simulated EL diagram for the emission from the neutral excited state S_1 as a

function of the bias voltage V_b and the electron injection barrier ϕ_e. The dominating excitation mechanism at each region is also schematically shown around the EL diagram. Here the hole injection barrier ϕ_h is set to be 1.1 eV. We note that due to the energy level broadening (not considered in our model), the electron transfer between the Fermi level of the substrate and the T_1 state could be bidirectional for H_2Pc/3ML-NaCl/Au(111), since the HOMO position in the *dI*/*dV* spectrum is close to the triplet energy. The above setting for ϕ_h is believed to properly account for the excitation at the positive bias voltage. Here we use solid black arrows for carrier injections (CI), dashed green arrows for inelastic electron scattering (IES), red arrows for photon emission, vertical dashed lines to illustrate level shifting due to charging/discharging, and pink wavy lines to connect transitions that occur simultaneously. The same annotations are adopted for other similar figures throughout the whole manuscript.

Supplementary Note 7.1.2 EL diagrams considering transient excited charged states

As shown in Supplementary Fig. 9(a), when excited at negative bias voltages, in addition to the neutral Q-band emission at ~1.81 eV (*i.e.*, Q_x), there is another sharp emission at the lower-energy side around 1.39 eV, which can be attributed to the emission from the D_1^+ doublet state of a cationic H_2Pc^+[27]. The onset voltage for such cationic emission is found to be about −1.46 V (Supplementary Fig. 9(b)). Such a simultaneous observation of both neutral and cationic emission suggests that the intermediate cationic state not only emerges as the ground state D_0^+ in the excitation sequence, but also has certain probabilities to be promoted to the excited state D_1^+, the latter can decay radiatively to produce cationic emission. Therefore, to better understand the excitation mechanism at negative bias for the H_2Pc/NaCl/Au(111) system, we also construct EL diagram by taking into account the transient excited cationic state (D_1^+) as well as its emission, with the corresponding excitation mechanisms for each region shown around the EL diagram (Supplementary Fig. 10). Nevertheless, we would like to note that since the cationic excited charged state D_1^+ is unlikely to be involved when excited at positive bias and no anionic emission was observed experimentally, the EL diagrams shown in main-text Fig. 3 and Supplementary Fig. 8 for the electroluminescence at positive voltages are still valid, though only the ground state D_0^- of the transient anionic state is considered in the simulation there.

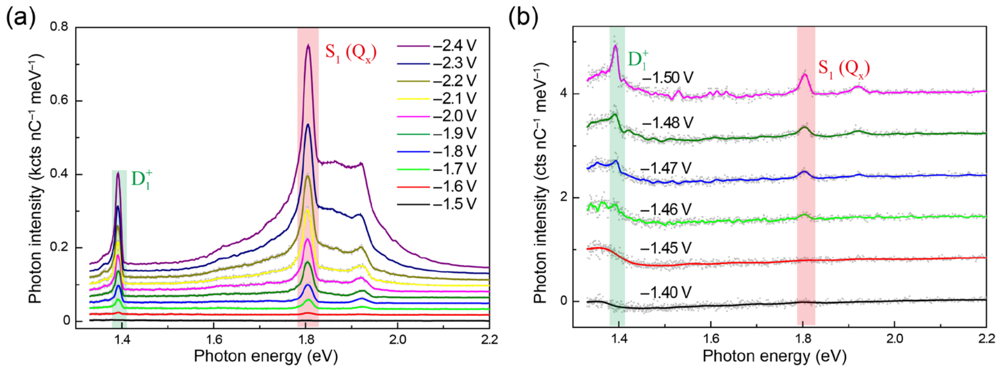

Supplementary Fig. 9: STML spectra for the H_2Pc/2ML-NaCl/Au(111) system. (a) STML spectra at different negative voltages from $V_b = -1.5$ V to -2.4 V (50 pA, 120 s). (b) STML spectra at negative bias voltages between $V_b = -1.4$ V and -1.5 V with larger tunneling currents and longer collection time (200 pA, 1200 s) to reveal the electroluminescence onset voltage. Spectral curves are offset for clarity. The emission peaks at ~1.81 eV and ~1.39 eV correspond to the emission from S_1 and D_1^+, respectively.

In the following, we focus our discussion on Region ④ in Supplementary Fig. 10(a) and Region ③ in Supplementary Fig. 10(b), since the electroluminescence intensities in other regions are probably too low to be detected experimentally. As illustrated in the excitation mechanism for Region ④ in Supplementary Fig. 10(a), to reach the cationic excited state D_1^+, the molecule is firstly excited to the spin-triplet state T_1. Then the electron in the “HOMO” of the T_1 state transfers to the lower-lying Fermi level of the tip, with its excess energy transferred to the electron in the “LUMO” of the T_1 state. This is obviously a many-body process, which can be achieved via the Coulomb interaction between the two electrons. We can estimate the onset voltage of Region ④ in Supplementary Fig. 10(a) as follows. The triplet excitation energy (~1.2 eV) is ~0.19 eV lower than the cationic excitation energy (~1.39 eV). According to the energy conservation law, to achieve the transition from T_1 to D_1^+, the Fermi level of the tip has to lie below the HOMO state by at least 0.19 eV. This simple argument suggests that the threshold voltage for exciting the molecule to D_1^+ is about −1.49 V considering the hole injection barrier of ~1.3 eV selected here according to the HOMO peak position experimentally observed, which agrees with the experimental data (Supplementary Fig. 10(b)) quite well. Moreover, we believe that the efficiency of this Auger-like process will increase with increasing bias voltages (*i.e.*, the Fermi level of the tip is lower), because at higher voltages the electron that leaves the molecule can donate more energy to the electron

remaining in the molecule. This might be the reason why the intensity of D_1^+ quickly grows with increasing bias voltage in the experiment.

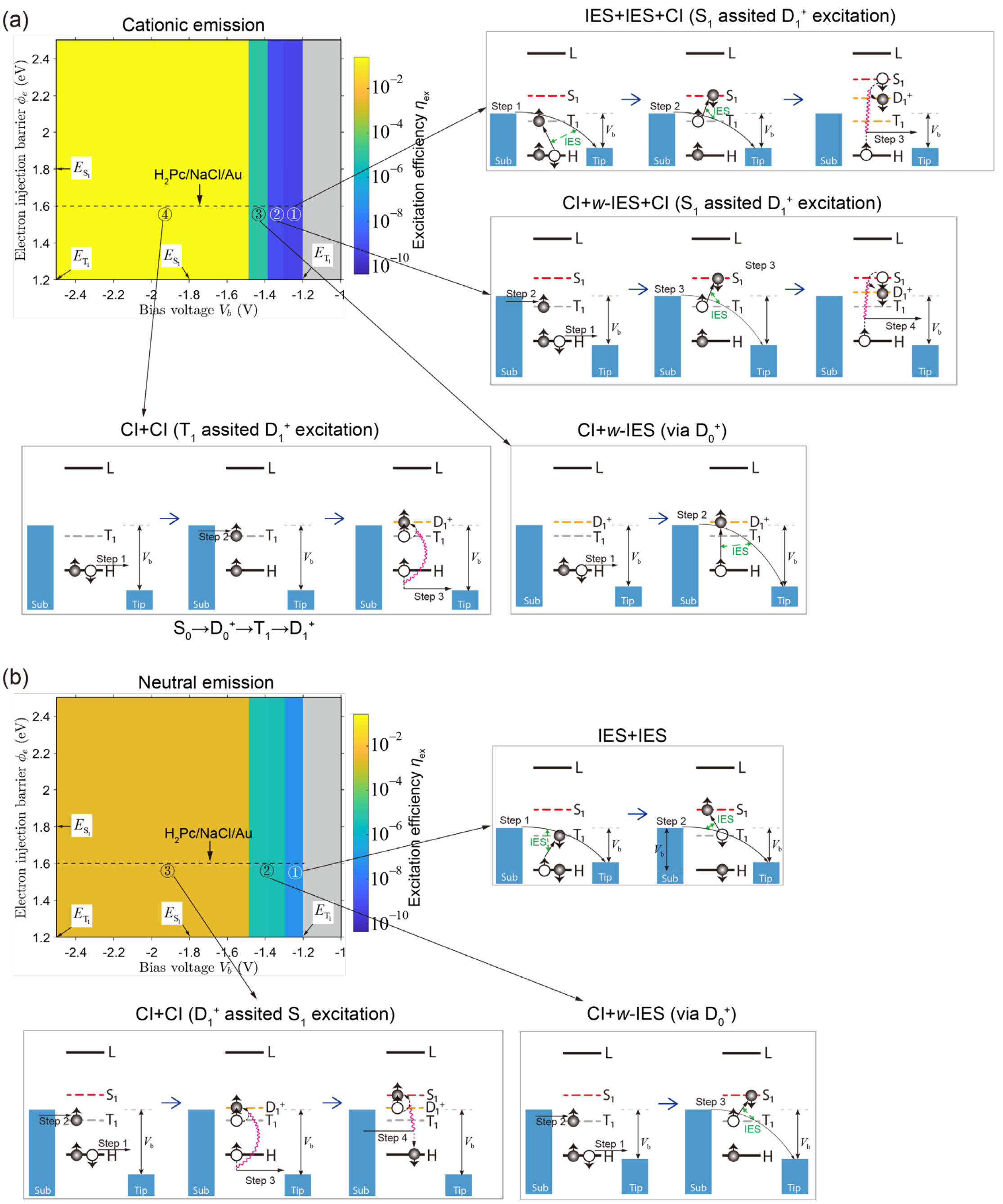

Supplementary Fig. 10: Simulated EL diagram considering transient excited charged states at negative bias voltages. (a,b) Electroluminescence from the cationic excited state D_1^+ (a) and neutral excited state S_1 (b) as a function of the bias voltage V_b and the electron injection barrier ϕ_e. Here the hole injection barrier ϕ_h is set to be 1.3 eV. We note that due to the energy level broadening, the electron transfer between the Fermi level of the substrate and the T_1 state is probably bidirectional for H_2Pc/3ML-NaCl/Au. The above setting for ϕ_h is believed to properly account for the excitation at the negative bias voltage.

After rationalizing the excitation mechanism of the light emission from the cationic state D_1^+, the neutral emission is easy to understand. As shown in the excitation sketch for Region ③ in Supplementary Fig. 10(b), once the molecule is excited to D_1^+, another electron can quickly tunnel into the "HOMO" of D_1^+, which changes the molecule to the neutral excited state S_1. Therefore, the intensity of the neutral emission is strongly correlated with the cationic emission, as can be seen in Supplementary Fig. 10. Note that the mechanism shown in region ③ of Supplementary Fig. 10(b) refers to both the upconversion and normal-bias electroluminescence. For the UCEL region at negative biases, both the IES+IES mechanism in region ① and the CI+*w*-IES mechanism in region ② shown in Supplementary Fig. 10(b) are very inefficient, yielding negligible S_1 emission when $V_b \leq -1.45$ V. The dominant mechanism for the UCEL phenomenon from –1.46 V to –1.81 V is believed to associate with another type of CI+CI mechanism that involves a transient excited state of the intermediate cation D_1^+. Specifically, to reach the S_1 state, the following four steps are required (see region ③ in Supplementary Fig. 10(b)). First, the electron in the HOMO tunnels to the tip (step 1), leaving behind a transient ground-state cation D_0^+ (in other words, a hole is injected into the HOMO); Second, the D_0^+ state can be transformed to the neutral T_1 state upon an electron injection from the substrate (step 2); Third, the electron in the "HOMO" of the T_1 state transfers to the lower-lying Fermi level of the tip, with its excess energy transferred to the electron in the "LUMO" of the T_1 state, inducing the molecule to the excited-state cation D_1^+ (step 3); Fourth, another electron in the substrate tunnels into the "HOMO" of D_1^+, changing the molecule to the neutral excited state S_1 (step 4). Nevertheless, presumably limited by the short lifetime of the transient excited state D_1^+, the UCEL intensity at the negative bias is weaker than that at the positive bias. Note that, to efficiently excite the molecule from D_1^+ to S_1, the tunneling rate from the substrate to the molecule $\gamma_{\mathrm{sub}\rightarrow\mathrm{mol}}$ has to be comparable to or larger than the spontaneous emission rate from D_1^+ to D_0^+. Therefore, an increase in the thickness of the NaCl layer will slow down $\gamma_{\mathrm{sub}\rightarrow\mathrm{mol}}$ and thus decrease the ratio of the S_1 emission intensity over that of D_1^+[28].

It should be noted in real experiments, the finite broadening of molecular levels (as can be inferred from the *dI*/*dV* peaks) should be considered. For instance, when assuming fixed energy-level positions $\phi_h < E_{T_1}$, electrons can tunnel from the molecular T_1 to the substrate but not vice versa. However, the experimental HOMO state in the *dI*/*dV* spectra expands from –1.1 V to –1.5 V,

which suggests that the actual HOMO is broadened. For H_2Pc with a triplet energy of ~1.2 eV, bidirectional electron tunneling between the Fermi level of the substrate and the T_1 state is possible. For simplicity, the energy-level broadening is not considered in our simulation. Instead, we modify slightly the electron (hole) injection barriers to capture the dominant excitation mechanisms. For example, we use ϕ_h = 1.1 eV to explain the abnormal UCEL at positive voltages in main-text Fig. 3, whereas ϕ_h is set to 1.3 eV in order to consider the cationic excited state in the electroluminescence at negative bias voltages here. Nevertheless, these mathematical treatments do not affect the essential physical picture of the EL diagram for the interested regions.

Supplementary Note 7.1.3 Comparison with previous STML studies for the "same" H_2Pc/3ML-NaCl/Au(111) system

We would like to note that different STML phenomena were reported for the "same" H_2Pc/3ML-NaCl/Au(111) system by Rai *et al.*[8], probably due to different adsorption configurations of H_2Pc molecules on NaCl and resultant different energy level alignments at the molecular interface. Unlike the anomalously bright UCEL behavior at 1.7 V with a photon intensity of ~5 kcts nC^{-1} meV^{-1} that is even stronger than the normal-bias intensity of ~1 kcts nC^{-1} meV^{-1} at 2.0 V, the work by Rai *et al.*[8] reports an upconversion intensity of ~0.4 kcts nC^{-1} meV^{-1} at 1.6 V that is weaker than the normal-bias emission intensity of ~2 kcts nC^{-1} meV^{-1} at 2.1 V. Such a difference in the emission behavior is hard to understand if the systems are really exactly the same. Nevertheless, by comparing the experimental differential conductance data, we notice that the HOMO positions in the *dI/dV* data are quite different in these two works, which may account for the distinct STML phenomena. We would like to note that the *dI/dV* features in our present work are consistent with those reported by Imai-Imada *et al.*[29] for the same H_2Pc/NaCl/Au(111) system. In our *dI/dV* data, the HOMO is peaked at around –1.3 V with an onset at about –1.1 V (see main-text Fig. 1c), while in Ref. 8, the HOMO peak is centered at around –2.3 V. Such a large difference in the HOMO position is probably responsible for the distinct STML phenomena reported, in particular for the absence of the anomalously bright UCEL phenomenon in Ref. 8. Specifically, as shown in main-text Fig. 2b, for the CI+CI UCEL mechanism to operate at positive bias, the T_1 triplet state (with an energy of ~1.2 eV) must lie above the Fermi level of the substrate for carrier injection to occur via step 3. While this requirement for the energy level alignment is satisfied in our present system, it breaks down in

the case of Ref. 8 where the HOMO lies deep below the Fermi level of the substrate. Nevertheless, it is still not clear why the HOMO position is quite different for the "same" system. Perhaps, local perturbations due to defects might be possible reasons for such differences in the HOMO positions, as discussed in the PhD thesis of Grewal for the PtPc/NaCl/Au(111) system (see pages 49–51 in the PhD thesis[9]).

Supplementary Note 7.2 H_2Pc/3ML-NaCl/Ag(100)

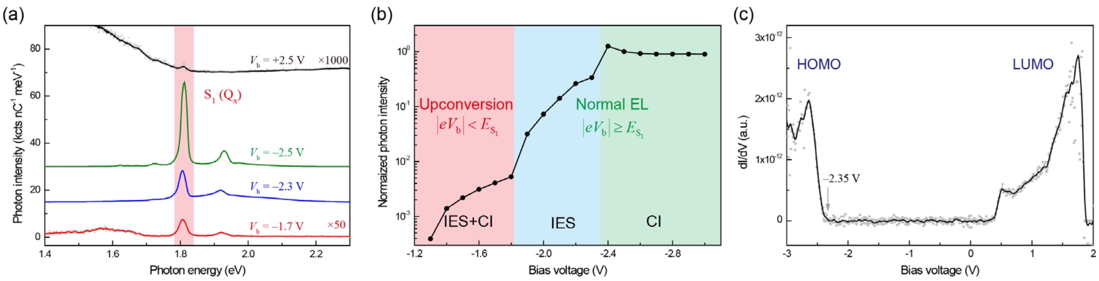

Supplementary Fig. 11: Electroluminescence for H_2Pc/3ML-NaCl/Ag(100). (a) STML spectra at V_b = –1.7, –2.3, –2.5 V (20 pA, 60 s) and +2.5 V (100 pA, 180 s). Spectral curves are offset for clarity. (b) Normalized bias-dependent electroluminescence intensity integrated over the Q_x peak. (c) Differential conductance (dI/dV) of a single H_2Pc molecule on 3ML-NaCl/Ag(100), set point: –2.5 V, 5 pA.

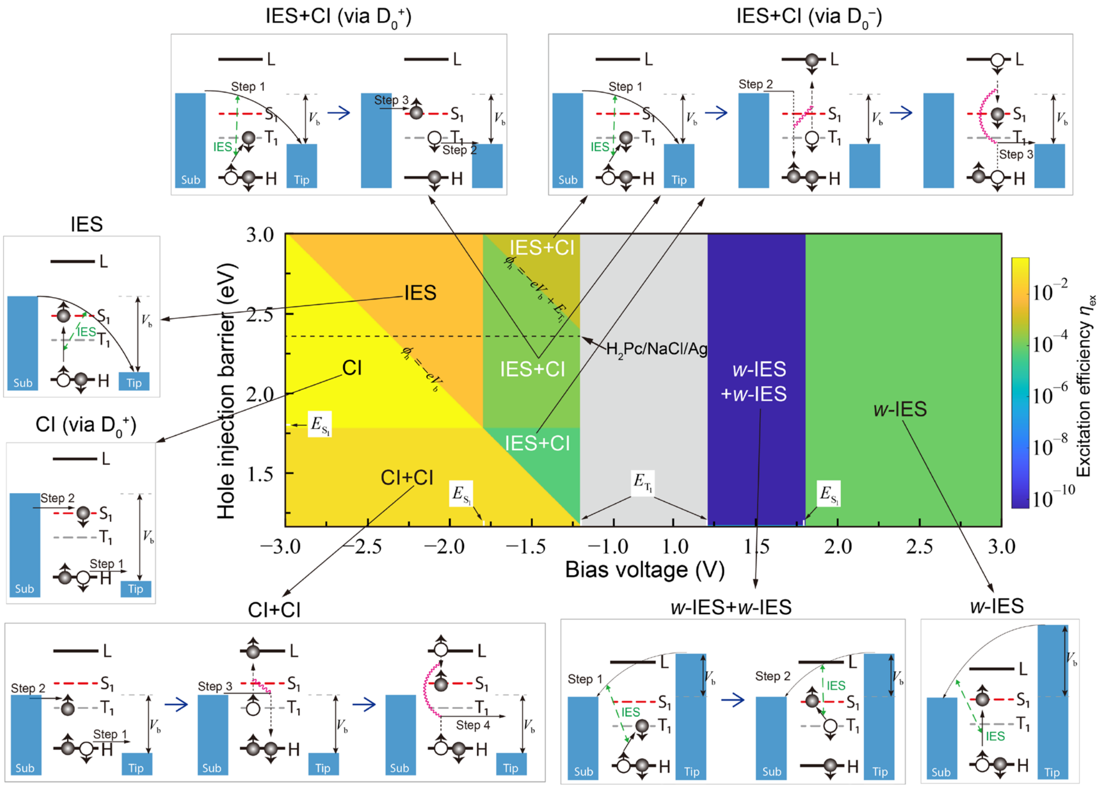

Supplementary Fig. 12: Simulated EL diagram from the neutral excited state S_1 as a function of the bias voltage V_b and the electron injection barrier ϕ_e. The excitation mechanism at each region is also schematically shown around the EL diagram. Here the electron injection barrier ϕ_e is set to be 0.5 eV

to simulate the condition for H_2Pc/3ML-NaCl/Ag(100).

The UCEL phenomenon for the H_2Pc/2ML-NaCl/Ag(100) system has been reported in Ref. 7. Here, we show typical experimental data for H_2Pc/3ML-NaCl/Ag(100) in Supplementary Fig. 11. Supplementary Fig. 12 schematically illustrates EL diagram and corresponding excitation mechanisms. The horizontal dashed line shown in Supplementary Fig. 12 corresponds to special case of the H_2Pc/3ML-NaCl/Ag(100) system. The excitation processes along the dashed line have been discussed in detail in Ref. 7. Depending on the value of the hole injection barrier ϕ_h, the IES+CI mechanism in the plot splits into three regions with different molecular excitation efficiencies via either a transient cationic state or a transient anionic state or both.

Supplementary Note 8. Bias-dependent STML spectra for PtPc/NaCl/Au(111)

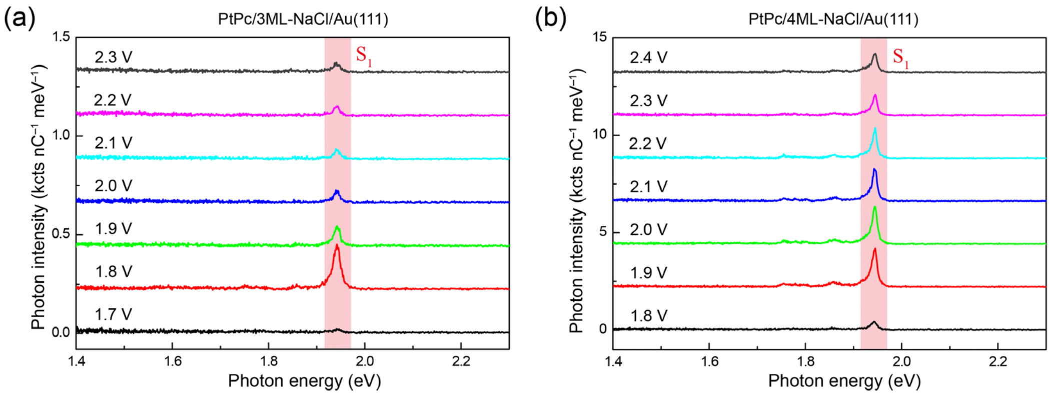

Supplementary Fig. 13: Bias-dependent STML spectra for PtPc/NaCl/Au(111) with two different thicknesses of NaCl layers. (a) Bias-dependent STML spectra for PtPc/3ML-NaCl/Au(111) measured at V_b = 1.7–2.3 V (100 pA, 10 s). (b) Bias-dependent STML spectra for PtPc/4ML-NaCl/Au(111) collected at V_b = 1.8–2.4 V (10 pA, 30 s). Spectral curves are offset for clarity. We note that the PtPc molecule becomes unstable at lower bias voltages when the tip gets closer to the molecules. By comparing with the STML spectral data in main-text Fig. 1b, the UCEL intensity for PtPc/3ML-NaCl/Au(111) at V_b = 1.8 V appears weaker than the UCEL intensity for H_2Pc/3ML-NaCl/Au(111) at V_b = 1.7 V.

References

1 Yang, B. *et al.* Sub-nanometre resolution in single-molecule photoluminescence imaging. *Nat. Photon.* **14**, 693-699 (2020).

2 Imada, H. *et al.* Single-molecule laser nanospectroscopy with micro–electron volt energy resolution. *Science* **373**, 95-98 (2021).

3 Rosławska, A. *et al.* Mapping Lamb, Stark, and Purcell effects at a chromophore-picocavity junction with hyper-resolved fluorescence microscopy. *Phys. Rev. X* **12**, 011012 (2022).

4 Doppagne, B. *et al.* Single-molecule tautomerization tracking through space-and time-resolved fluorescence spectroscopy. *Nat. Nanotechnol.* **15**, 207-211 (2020).

5 Peters, P.-J. *et al.* Quantum coherent multielectron processes in an atomic scale contact. *Phys. Rev. Lett.* **119**, 066803 (2017).

6 McVie, J., Sinclair, R. S. & Truscott, T. G. Triplet states of copper and metal-free phthalocyanines. *J. Chem. Soc., Faraday Trans. 2* **74**, 1870-1879 (1978).

7 Chen, G. *et al.* Spin-Triplet-Mediated Up-Conversion and Crossover Behavior in Single-Molecule Electroluminescence. *Phys. Rev. Lett.* **122**, 177401 (2019).

8 Rai, V. *et al.* Boosting light emission from single hydrogen phthalocyanine molecules by charging. *Nano Lett.* **20**, 7600-7605 (2020).

9 Grewal, A. Single molecule fluorescence and phosphorescence studies using a scanning tunneling microscope. (EPFL, 2022).

10 Hipps, K. & Mazur, U. Inelastic electron tunneling: an alternative molecular spectroscopy. *J. Phys. Chem.* **97**, 7803-7814 (1993).

11 Bredas, J.-L. Mind the gap! *Mater. Horiz.* **1**, 17-19 (2014).

12 Rosławska, A. *et al.* Single charge and exciton dynamics probed by molecular-scale-induced electroluminescence. *Nano Lett.* **18**, 4001-4007 (2018).

13 Obolda, A. *et al.* Triplet–polaron-interaction-induced upconversion from triplet to singlet: a possible way to obtain highly efficient OLEDs. *Adv. Mater.* **28**, 4740-4746 (2016).

14 Moth-Poulsen, K. & Bjørnholm, T. Molecular electronics with single molecules in solid-state devices. *Nat. Nanotechnol.* **4**, 551-556 (2009).

15 Zhou, J., Wang, K., Xu, B. & Dubi, Y. Photoconductance from exciton binding in molecular junctions. *J. Am. Chem. Soc.* **140**, 70-73 (2018).

16 Doppagne, B. *et al.* Vibronic spectroscopy with submolecular resolution from STM-induced electroluminescence. *Phys. Rev. Lett.* **118**, 127401 (2017).

17 Chen, G., Li, X. G., Zhang, Z. Y. & Dong, Z. C. Molecular hot electroluminescence due to strongly enhanced spontaneous emission rates in a plasmonic nanocavity. *Nanoscale* **7**, 2442-2449 (2015).

18 Zhang, Y., Zelinskyy, Y. & May, V. Plasmon-enhanced electroluminescence of a single molecule: A theoretical study. *Phys. Rev. B* **88**, 155426 (2013).

19 Hansma, P. K. Inelastic electron tunneling. *Phys. Rep.* **30**, 145-206 (1977).

20 Svatek, S. A. *et al.* Triplet excitation and electroluminescence from a supramolecular monolayer embedded in a boron nitride tunnel barrier. *Nano Lett.* **20**, 278-283 (2020).

21 Seldenthuis, J. S., van der Zant, H. S., Ratner, M. A. & Thijssen, J. M. Electroluminescence spectra in weakly coupled single-molecule junctions. *Phys. Rev. B* **81**, 205430 (2010).

22 Sanche, L. Low-energy electron scattering from molecules on surfaces. *J. Phys. B: At. Mol. Opt.*

Phys. **23**, 1597 (1990).

23 Farrukh, A. *et al.* Bias-polarity dependent electroluminescence from a single platinum phthalocyanine molecule. *Chin. J. Chem. Phys.* **34**, 87-94 (2020).

24 Qiu, X. H., Nazin, G. V. & Ho, W. Vibrationally resolved fluorescence excited with submolecular precision. *Science* **299**, 542-546 (2003).

25 Miwa, K. *et al.* Many-body States Description of Single-molecule Electroluminescence Driven by Scanning Tunneling Microscope. *Nano Lett.* **19**, 2803-2811 (2019).

26 Zhu, J. Z., Chen, G., Ijaz, T., Li, X. G. & Dong, Z. C. Influence of an atomistic protrusion at the tip apex on enhancing molecular emission in tunnel junctions: A theoretical study. *J. Chem. Phys.* **154**, 214706 (2021).

27 Doležal, J. *et al.* Evidence of trion-libron coupling in chirally adsorbed single molecules. *Nat. Commun.* **13**, 6008 (2022).

28 Doppagne, B. *et al.* Electrofluorochromism at the single-molecule level. *Science* **361**, 251-254 (2018).

29 Imai-Imada, M. *et al.* Energy-level alignment of a single molecule on ultrathin insulating film. *Phys. Rev. B* **98**, 201403 (2018).
